# Invasive pulmonary mucormycosis and aspergillosis in a patient with decompensated hepatic cirrhosis

**DOI:** 10.1016/j.mmcr.2018.03.004

**Published:** 2018-03-06

**Authors:** Jon G. Persichino, Argun D. Can, Tam T. Van, Michele N. Matthews, Scott G. Filler

**Affiliations:** a Harbor-UCLA Medical Center, Division of Infectious Diseases, 1000 West Carson Street, Torrance, CA 90509, USA; b Harbor-UCLA Medical Center, Division of Respiratory & Critical Care Physiology & Medicine, 1000 West Carson Street, Torrance, CA 90509, USA; c Harbor-UCLA Medical Center, Department of Pathology, 1000 West Carson Street, Torrance, CA 90509, USA

**Keywords:** Invasive, Pulmonary, Mucormycosis, Aspergillosis, Hepatic cirrhosis

## Abstract

Invasive pulmonary mucormycosis and aspergillosis are rare, life-threatening fungal infections. Most documented cases have been reported in non-cirrhotic patients with diabetes mellitus, neutropenia, or treatment with corticosteroids. The prevalence of each infection is low among patients with hepatic cirrhosis. We report the first likely case of combined invasive pulmonary mucormycosis and aspergillosis in a male with decompensated hepatic cirrhosis. This report also highlights the first non-diabetic case of invasive pulmonary mucormycosis with decompensated hepatic cirrhosis.

## 1 Introduction

Mucormycosis and Aspergillosis are rare, life-threatening fungal infections with mortality rates reported to be over 50% despite surgical debridement and antifungal therapy [1], [2]. *Rhizopus arrhizus*, which is responsible for 70% of all cases of mucormycosis, is associated with a number of clinical diseases (rhino-orbital-cerebral, cutaneous, gastric, and pulmonary mucormycosis) in adults [1]. This fungal infection is particularly recognized worldwide by the healthcare community due to its highly pathogenic nature, which is characterized by rapid tissue destruction and invasion across tissue planes [3]. The major risk factors for mucormycosis include uncontrolled diabetes mellitus with ketoacidosis, hematological malignancy, stem cell and solid organ transplantations, iron chelation therapy with deferoxamine, and corticosteroid usage [3]. The diagnosis of mucormycosis is usually made by the identification of causative fungal organisms by histopathological analysis of tissue specimens from patients with suggestive signs and symptoms [3]. Cultures are only occasionally positive [3]. Initial treatment of mucormycosis typically requires early aggressive surgical debridement of infected tissues, combined with administration of amphotericin B deoxycholate (Amb) or liposomal amphotericin B (L-AmB) [3].

*Aspergillus fumigatus* is responsible for over 70% of all cases of invasive aspergillosis [4]. Invasive aspergillosis usually occurs in immunosuppressed patients such as those receiving chronic corticosteroid treatment or with prolonged neutropenia from malignancy [2], [4]. Other risk factors for infection include chronic obstructive pulmonary disease and hepatic cirrhosis [2]. The diagnosis of proven, probable, or possible invasive aspergillosis is made by the combination of host status, imaging and mycological findings [5]. The optimal treatment of aspergillosis is administration of voriconazole or isavuconazole, which must be used with caution in patients with hepatic cirrhosis [2], [4].

Here, we report a patient with decompensated hepatic cirrhosis who had combined invasive pulmonary mucormycosis and aspergillosis. To our knowledge, this is the first report of invasive pulmonary mucormycosis in a decompensated cirrhotic patient who did not have concomitant diabetes mellitus.

## 2 Case

A 58-year old homeless male was found unresponsive in a parking lot and was taken by ambulance to our emergency department (ED). The patient was minimally responsive and able to state only his name; no additional history could be obtained. Initial vital signs in the ED demonstrated a temperature of 37.1 °C, heart rate of 157 beats per minute, respiratory rate of 35 per minute, oxygen saturation of 78% while breathing 15 l of oxygen per minute via face mask, and blood pressure of 80/49 mmHg. On examination, he was ill appearing, icteric, and in respiratory distress. The patient had tachycardia, but no cardiac murmurs. Rales were heard at both lung bases. The entire right lower extremity was erythematous and there was extensive necrosis of the soft tissues extending from the hindfoot to the proximal thigh.

**Fig. 1:**
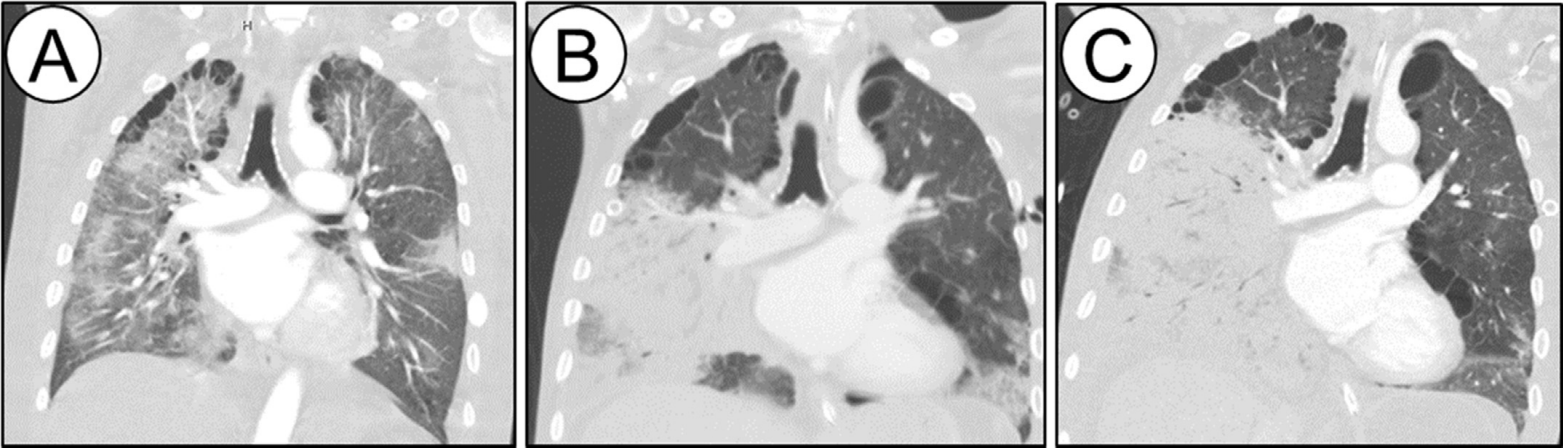
Coronal images (slices) from CT of chest scans on days 1 (A), 16 (B), and 23 (C) of hospitalization demonstrating the progression of the infiltrates in the right lung.

Pertinent laboratory studies on initial presentation revealed a white blood cell count of 19.2 × 10^3^ cells/µm^3^, platelets of 19 × 10^3^ cells/µm^3^, bicarbonate of 21 mEq/L, blood urea nitrogen of 67 mg/dL, creatinine of 1.23 g/dL, random glucose of 120 mg/dL, total bilirubin of 10.3 mg/dL, direct bilirubin of 6.1 mg/dL, alanine transaminase of 66 U/L, aspartate transaminase of 116 U/L, albumin of 1.4 g/dL, prothrombin time of 34, international normalized ratio of 3.3, partial thromboplastin time of 47 U/L, creatine phosphokinase of 851 U/L, and lactate of 3.3 mg/dL. A portable chest radiograph (CXR) showed patchy bibasilar infiltrates. Computed tomography (CT) of the chest revealed bilateral ground-glass opacities and infiltrates that were more extensive in the right lung (Fig. 1A).

The patient was treated with aggressive intravenous (IV) fluid hydration and vasopressors (norepinephrine, vasopressin, and epinephrine) for septic shock. He underwent tracheal intubation for acute hypoxic respiratory failure. The patient received empiric antibiotic treatment with IV vancomycin, piperacillin-tazobactam, and clindamycin. The patient was taken to the operating room where a right above-the-knee amputation was performed for his necrotizing soft tissue infection on day 0.

The patient's postoperative course was complicated by refractory septic shock requiring multiple vasopressors, acute anuric renal failure necessitating hemodialysis, bilateral chest tube drainage of pleural effusions, worsening liver failure, and progressive hypoxia requiring mechanical ventilation. On day+ 2, the patient underwent surgical disarticulation of his right hip to remove residual necrotic infected tissue. The intraoperative wound cultures grew methicillin-sensitive *Staphylococcus aureus* and *Proteus penneri*. On day+ 4, 1/4 bottles of the blood cultures grew *Aerococcus* species and *Staphylococcus epidermidis*. All four organisms were sensitive to vancomycin and/or piperacillin-tazobactam. No fungal organisms were seen on histopathological analysis of infected tissue and none were isolated from the cultured material.

The patient continued to deteriorate after surgery. On day+ 10, given the continued refractory septic shock and bibasilar patchy infiltrates seen on CXR, a bronchoalveolar lavage (BAL) was performed, and thick frothy respiratory secretions with pus were seen. Gram stain and bacterial culture of the BAL specimen were negative, and Xpert^®^ MTB/RIF (Cepheid, Sunnyvale, CA) PCR of the sputum was negative for tuberculosis. Cytopathology was not performed.

**Fig. 2:**
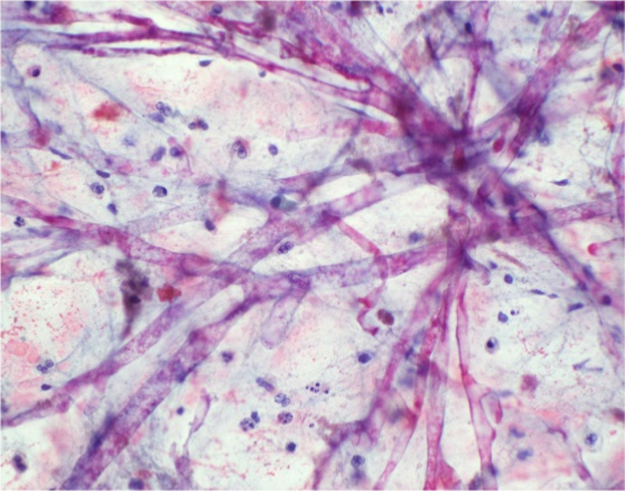
Nonseptate hyphae from BAL cytology specimen (original magnification X 400).

On day+ 12, an infectious diseases consultation was obtained. On initial evaluation, it was noted that the patient had a positive serum 1,3 β-D glucan (> 500, normal < 60). Empiric antifungal treatment with IV micafungin was initiated. Serum galactomannan, cryptococcal antigen, and coccidiomycosis complement fixation tests were negative, as was the urine histoplasma antigen test. On day+ 16, a repeat CT scan of the chest revealed worsening of right lung patchy infiltrates (Fig. 1B). A repeat BAL was performed on day+ 18, and thick frothy respiratory secretions were again seen. A bronchoscopic biopsy was not performed because of the patient's coagulopathy and thrombocytopenia. Nonseptate hyphae were seen on cytologic analysis of the repeat BAL specimen (Fig. 2). The *Aspergillus* galactomannan assay of the BAL fluid was also positive (6.78, normal < 0.5), and IV voriconazole was initiated on day+ 19. A rapidly growing mold grew in cultures of both the BAL sample and a swab from the right chest tube site; the specimens were sent to an outside reference laboratory for identification.

The patient continued to deteriorate. On day+ 21, when the total bilirubin increased to 23.5 mg/dL, the micafungin and voriconazole were discontinued and IV L-AmB (5 mg/kg daily) was initiated. On day+ 23, a CT scan of the chest showed worsening of the infiltrates in the right lung (Fig. 1C). In addition, a second mold grew in BAL fungal culture. This mold had septate hyphae and conidia, but was not sent for identification. The patient expired on day+ 25. Four days later, the rapidly growing mold from the BAL and right chest tube insertion site was identified as *R. arrhizus*, indicating the presence of invasive pulmonary mucormycosis.

## 3 Discussion

The data indicate that this patient had both invasive pulmonary mucormycosis and aspergillosis. The diagnosis of mucormycosis was made by the isolation of *R. arrhizus* from cultures of the BAL fluid and chest tube insertion site, and the finding of broad non-septate hyphae on the BAL cytology specimen. The patient also met the diagnostic criteria for probable invasive pulmonary aspergillosis based on his clinical presentation, the growth from the BAL culture of a second fungus with septate hyphae and conidia that resembled *Aspergillus* based on colony and microscopic morphologies, and positive BAL galactomannan and serum 1,3 B-D glucan tests [5]. Of note, neither test should be positive in patients with mucormycosis [6]. However, because no formal identification of the second mold from BAL fungal culture was performed, we cannot classify this case as proven aspergillosis [5]. The patient most likely acquired both organisms from the natural environment given his homelessness.

This is the first report of a case of combined invasive pulmonary mucormycosis and aspergillosis in a patient with decompensated cirrhosis. The patient did not have any of the traditional risk factors for both infections [2], [3], [4]. There was no evidence of diabetes mellitus given the patient's normal random glucose levels upon initial presentation and during hospitalization. The patient was never neutropenic, had only transient lactic acidosis, and did not receive prolonged corticosteroids.

Liver cirrhosis was the most likely contributing risk factor for both infections in our patient. Invasive aspergillosis is well-described in patients with decompensated liver cirrhosis [2]. Immune dysfunction is a known complication of liver cirrhosis [6]. A decline in both humoral and cell-mediated immunity is a risk factor for bacterial and fungal infections in patients with liver disease, especially in Child-Pugh (CP) class B and C cirrhosis [7], [8], [9]. The patient was also at higher risk for pulmonary aspergillosis given his positive emphysematous image findings (Fig. 1A-C) suggestive of chronic obstructive pulmonary disease [2]. Furthermore, the patient was in an iatrogenic iron overload state after receiving 22 units of packed red blood cells for anemia during his early-to-mid hospitalization course. This iron overload state can enhance fungal growth and virulence, possibly contributing to his clinical deterioration [3]. Another potential explanation to consider in our case was the early usage of voriconazole for aspergillosis. Animal models have demonstrated that voriconazole exposure increases virulence of *R. arrhizus* strains and mortality rates in pulmonary disease [10], [11].

Ultimately, our patient died from his invasive pulmonary mucormycosis and probable aspergillosis complicated by refractory septic shock, multi-organ failure, and severe immune dysfunction from decompensated liver cirrhosis. He also did not receive timely administration of L-Amb, the recommended drug of choice, for invasive pulmonary mucormycosis as this was an unexpected diagnosis given the rarity of this infection in decompensated liver cirrhosis.

To our knowledge, this is also the first case report of invasive pulmonary mucormycosis in a decompensated cirrhotic patient who did not have concomitant diabetes mellitus. The presenting and pertinent demographic and clinical characteristics of prior published case reports of invasive mucormycosis in patients with liver cirrhosis are shown in Table 1. Overall, these cases depict life-threatening infections associated with CP class C cirrhosis (14), diabetes mellitus (12), viral hepatitis C infection (9), rhino-orbital (8) and rhino-orbital-cerebral (7) involvements, CP class B cirrhosis (6), and alcohol use (6). The most intriguing findings are the vast disproportionate number of pulmonary (2) to rhino-orbital/rhino-orbital-cerebral (15) infections, no reports of infected cases associated with CP class A cirrhosis, and all cases with CP class C cirrhosis died despite IV Amb and surgical treatments. Our case differed from the 2 prior published cases (3 & 22) with pulmonary involvement in that there were no other risk factors of diabetes mellitus and chronic steroid usage as well as no association with CP class B cirrhosis.

**Table 1:** Demographics and clinical characteristics of reported cases of invasive mucormycosis in patients with liver cirrhosis.

**Case**	**Age- years**	**Gender**	**Cause of liver disease**	**Child Pugh class**	**Other risk factors**	**Location of infection**	**Treatment**	**Outcome**	**References**
1	44	Female	NR	B	None	RO	None	Died	[12]
2	63	Female	NR	NR	DM	ROC	None	Died	[13]
3	44	Male	ETOH	NR	DM	Pulmonary	AmB/Surgery	Alive	[14]
4	53	Male	HCV	B	DM	ROC	None	Died	[12]
5	58	Female	NR	C	DM	ROC	AmB	Died	[12]
6	39	Male	HCV	B	DM	ROC	AmB/Surgery	Died	[12]
7	57	Male	HCV	C	None	RO	AmB	Died	[12]
8	55	Male	HBV	C	None	RO	None	Died	[12]
9	15	Female	AIH	C	DM Steroids	RO	None	Died	[12]
10	53	Male	HCV	C	DM	ROC	AmB/Surgery	Died	[12]
11	35	Male	HCV	C	DM	RO	AmB	Died	[12]
12	38	Female	ETOH	C	Steroids	Cutaneous	Surgery	Died	[12]
13	63	Female	HCV	B	None	RO	AmB/Surgery	Alive	[12]
14	42	Male	HBV	C	None	RO	AmB	Died	[12]
15	59	Female	ETOH	C	None	ROC	None	Died	[12]
16	65	Male	HCV	B	DM	RO	AmB/Surgery	Alive	[12]
17	47	Male	HCV	C	DM	Gastric	AmB	Died	[12]
18	48	Female	ETOH	NR	None	Cutaneous	Surgery	Died	[12]
19	25	Female	AIH	C	Steroids	Cutaneous	AmB/Surgery	Died	[12]
20	55	Male	ETOH	NR	None	Gastric	AmB/Surgery	Alive	[15]
21	55	Female	AIH	C	DM Steroids	Gastric	AmB	Died	[16]
22	68	Woman	HCV	B	DM	Pulmonary	AmB/Surgery	Died	[17]
23	28	Male	ETOH	C	None	ROC	AmB	Died	[18]
24	58	Male	NR	C	None	Pulmonary	AmB	Died	This case

Abbreviations: NR, not reported; RO, rhino-orbital; ROC, rhino-orbital-cerebral; DM, diabetes mellitus; ETOH, alcohol; AmB, amphotericin B; HCV, Hepatitis C Virus; HBV, Hepatitis B Virus; AIH, autoimmune hepatitis.

In conclusion, this case report highlights the need for healthcare professionals to be aware that decompensated liver cirrhosis is considered a risk factor for both invasive pulmonary mucormycosis and aspergillosis.
